# Trypanosomatid-Caused Conditions: State of the Art of Therapeutics and Potential Applications of Lipid-Based Nanocarriers

**DOI:** 10.3389/fchem.2020.601151

**Published:** 2020-11-26

**Authors:** Giuliana Muraca, Ignacio Rivero Berti, María L. Sbaraglini, Wagner J. Fávaro, Nelson Durán, Guillermo R. Castro, Alan Talevi

**Affiliations:** ^1^Laboratory of Bioactive Research and Development (LIDeB), Department of Biological Sciences, Faculty of Exact Sciences, University of La Plata (UNLP), La Plata, Argentina; ^2^Administración Nacional de Medicamentos, Alimentos y Tecnología Médica (ANMAT), Buenos Aires, Argentina; ^3^Laboratorio de Nanobiomateriales, Centro de Investigación y Desarrollo en Fermentaciones Industriales (CINDEFI), Departamento de Química, Facultad de Ciencias Exactas, Universidad Nacional de La Plata (UNLP) -CONICET (CCT La Plata), La Plata, Argentina; ^4^Laboratory of Urogenital Carcinogenesis and Immunotherapy, Department of Structural and Functional Biology, Institute of Biology, University of Campinas (UNICAMP), Campinas, Brazil; ^5^Nanomedicine Research Unit (Nanomed), Federal University of ABC (UFABC), Santo André, Brazil

**Keywords:** Chagas, leishmaniasis, human African trypanosomiasis, lipid nanoparticles, liposomes, solid lipid nano particles, nanoestructed lipid carrier, nanoparticle

## Abstract

Trypanosomatid-caused conditions (African trypanosomiasis, Chagas disease, and leishmaniasis) are neglected tropical infectious diseases that mainly affect socioeconomically vulnerable populations. The available therapeutics display substantial limitations, among them limited efficacy, safety issues, drug resistance, and, in some cases, inconvenient routes of administration, which made the scenarios with insufficient health infrastructure settings inconvenient. Pharmaceutical nanocarriers may provide solutions to some of these obstacles, improving the efficacy–safety balance and tolerability to therapeutic interventions. Here, we overview the state of the art of therapeutics for trypanosomatid-caused diseases (including approved drugs and drugs undergoing clinical trials) and the literature on nanolipid pharmaceutical carriers encapsulating approved and non-approved drugs for these diseases. Numerous studies have focused on the obtention and preclinical assessment of lipid nanocarriers, particularly those addressing the two currently most challenging trypanosomatid-caused diseases, Chagas disease, and leishmaniasis. In general, *in vitro* and *in vivo* studies suggest that delivering the drugs using such type of nanocarriers could improve the efficacy–safety balance, diminishing cytotoxicity and organ toxicity, especially in leishmaniasis. This constitutes a very relevant outcome, as it opens the possibility to extended treatment regimens and improved compliance. Despite these advances, last-generation nanosystems, such as targeted nanocarriers and hybrid systems, have still not been extensively explored in the field of trypanosomatid-caused conditions and represent promising opportunities for future developments. The potential use of nanotechnology in extended, well-tolerated drug regimens is particularly interesting in the light of recent descriptions of quiescent/dormant stages of *Leishmania* and *Trypanosoma cruzi*, which have been linked to therapeutic failure.

## Introduction

**Figure 1:**
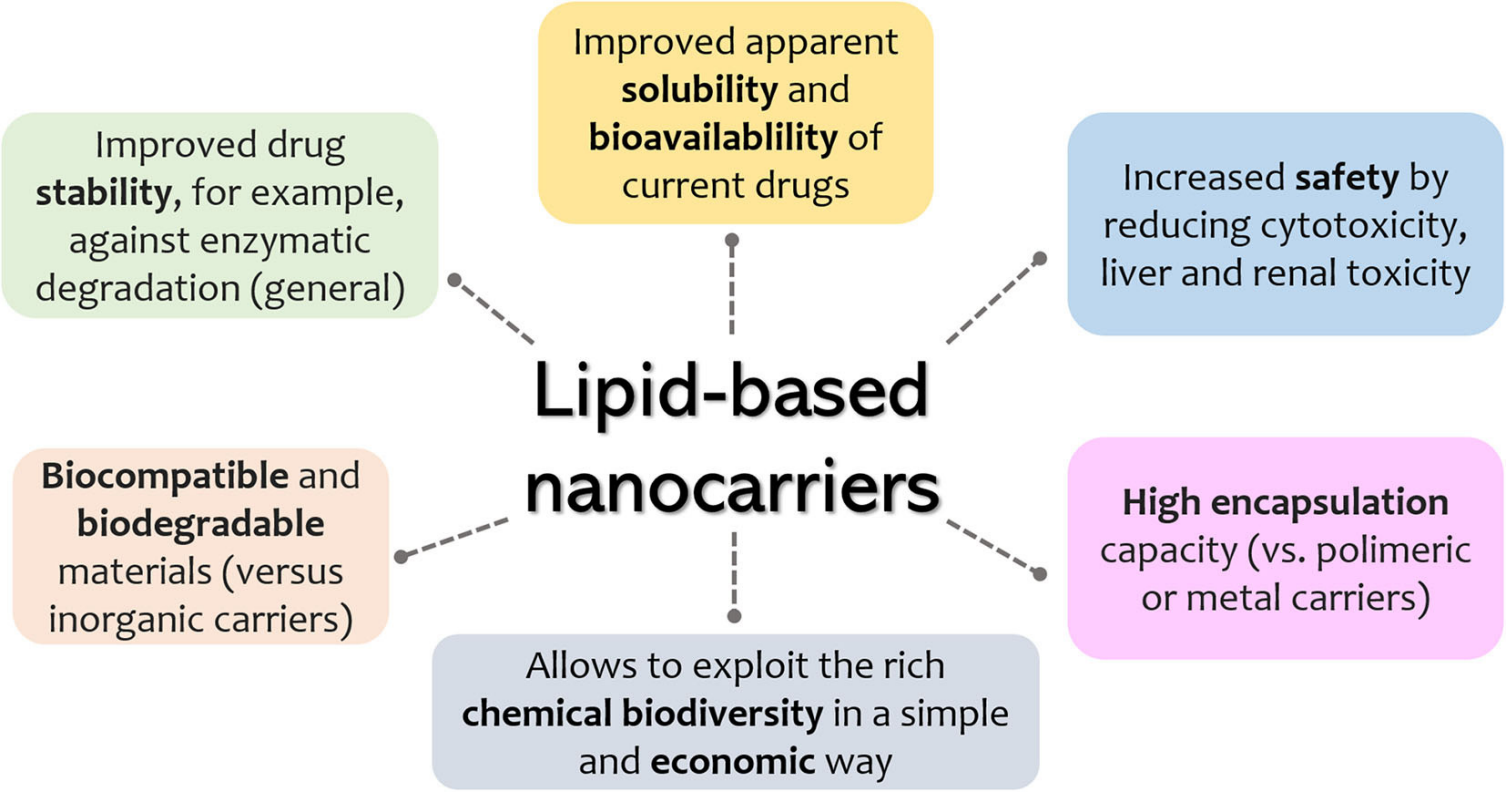
General advantages of lipid-based nanosystems.

Since the first description of liposomes in the mid-1960s, lipid-based pharmaceutical nanocarriers have made their way to the market and have undergone substantial evolution (Puri et al., 2009; Akbarzadeh et al., 2013). Their applications in the medical field include drug delivery, imaging molecules, decoration with ligands for site-specific targeting, and formulation with destabilizing lipid systems that allow for on-demand drug release. A relevant parameter of an ideal drug vehicle is the absence of toxicity (both for the patient and the environment) of the carrier itself or its by-products. Among the wide array of nanomaterials, lipid-based systems are undoubtedly among the safest ones (Puri et al., 2009). Later generations of lipid-based drug delivery systems include solid lipid nanoparticle (SLN), nanostructured-lipid carriers (NLCs), and, more recently, lipid–polymer hybrid systems (Puri et al., 2009; Mukherjee et al., 2019; Liu et al., 2020) and quatsomes (Ferrer-Tasies et al., 2013). It is expected that such drug carriers will allow for higher control over drug release, enhanced stability, and increased circulation times. Some general advantages of lipid nanosystems are schematically summarized in Figure 1.

Trypanosomatid-caused human conditions, namely, Chagas disease, leishmaniasis, and human African trypanosomiasis (HAT), are considered as neglected infectious diseases that have been listed as high-priority diseases by the World Health Organization (WHO), needing innovative and increased disease management (Parthasarathy and Kalesh, 2020). The real amount of infected people is possibly miscalculated as available figures are often extrapolation of data from incomplete epidemiological surveys. Also, the situation worsened by social and military conflicts and because of undiagnosed or unreported patients living in rural areas with limited access to health system facilities (Malvy and Chappuis, 2011). In 2010, it was estimated that these conditions affected about 27 million people worldwide and caused ~150,000 annual deaths (Nussbaum et al., 2010). Since then, depending on the case, advance and retreats have been registered. For instance, a 90% drop in the number of African trypanosomiasis cases has been realized between 2009 and 2018 (World Health Organization, 2020a), and the first all-oral medication for such condition has recently been launched (Deeks, 2019). On the other hand, a jump of 26.9% in leishmaniasis prevalence was observed between 2006 and 2016 (GBD 2016 Disease Injury Incidence Prevalence Collaborators, 2017). As no vaccine is available, treatment solely depends on chemotherapy. Specifically, in the case of Chagas disease and leishmaniasis, available treatment options are dramatically limited. Among disadvantages of much of the available therapeutic arsenal, we might mention toxicity, emerging drug-resistance issues, high costs, limited efficacy, and inconvenient routes of administration (Patterson and Wyllie, 2014). Therefore, novel therapeutic options are urgently required to improve both accessibility and therapeutic output and control potential resistance issues. Interestingly, the existence of quiescent and dormant stages (characterized by low or no replication and diminished metabolic activity) has been described, respectively, in *Leishmania* and *Trypanosoma cruzi* (Berg et al., 2013; Kloehn et al., 2015; Jara et al., 2017, 2019; Sánchez-Valdez et al., 2018). Depending on the case, such forms may emerge spontaneously (Sánchez-Valdez et al., 2018) or as an adaptive response to stress conditions (e.g., drug pressure) (Berg et al., 2013; Jara et al., 2019), contributing to treatment failure. It has been suggested that these new resistance mechanisms, which add to classical genetic-based drug resistance, may be treated using current drugs in extended therapeutic regimens that extend beyond the dormancy potential (Sánchez-Valdez et al., 2018).

Here, we will briefly overview the trypanosomatid-caused human diseases, along with the strengths and limitations known chemotherapies to treat such conditions and recent or ongoing clinical trials. Later, we will review the recent advances on lipid nanocarriers encapsulating available and potential pharmacological agents against Chagas disease, sleeping sickness, and leishmaniasis. Noteworthy, other types of nanomaterials (e.g., polymeric systems and metal nanoparticles) have also been investigated (see, for instance, Ahmad et al., 2020; Nafari et al., 2020), although they fall outside the scope of the present review and article collection. Finally, some concluding remarks including possible future directions in the field will be included.

Other recent reviews on the topic have been published, including a comprehensive review on drug nanocarriers of different materials targeting trypanosomatid-caused diseases (Volpedo et al., 2019) and reviews exclusively focused on a given condition (Quezada et al., 2019; Saleem et al., 2019) or in specific drugs encapsulated in a diversity of nanocarriers (Arrúa et al., 2019).

## Literature Survey

We initially search in Scopus all the combinations of one of the following terms “Chagas disease,” “leishmaniasis,” “human African trypanosomiasis,” “Trypanosoma cruzi,” “*Trypanosoma brucei*,” “Leishmania” with one of the following terms “nanocarrier,” “nanolipid,” “solid lipid nanoparticles,” “nanostructured lipid carrier,” “nanosystem,” “drug delivery.” Two of the authors (GM and MLS) scrutinized the abstracts of the resulting articles and selected those related to the review scope. The search was conducted in Google Scholar and Scopus.

## Human African Trypanosomiasis

HAT is transmitted by the tsetse fly *Glossina* spp.; the etiologic agents are two subspecies of *Trypanosoma brucei* (World Health Organization, 2020a). The disease presents a first stage (also called hemolymphatic, or early stage), taking place when the parasite invades the bloodstream, and a second stage (meningoencephalitic, or late stage) linked to invasion of the patient's central nervous system (CNS) by the parasite. In humans, the disease presents in two forms, depending on the parasite subspecies involved. When the etiological agent is *T. brucei gambiense* (gHAT), the disease usually evolves without major signs or symptoms. Nevertheless, when symptoms emerge, the patient has often reached the advanced stage of the disease, compromising the CNS and narrowing treatment options. On the other hand, when the infection is caused by *T. brucei rhodesiense* (rHAT), the disease develops rapidly, with the first signs/symptoms being observed as soon as a few weeks after infection, also compromising the CNS (World Health Organization, 2020a).

### Human African Trypanosomiasis Treatment

Today, WHO recommends a diversity of treatment options against HAT, depending on the parasite subspecies involved and the evolution stage of the disease, including pentamidine, suramin, melarsoprol, eflornithine, nifurtimox, and fexinidazole.

Pentamidine appeared in 1940 and has since then been employed for the treatment of the early phase of gHAT. It is an aromatic diamidine that has numerous undesirable side effects such as disturbances of glucose homeostasis, leukopenia, and hypotension, as well as an inconvenient route of administration (intramuscular) (Yang et al., 2014; Sbaraglini et al., 2016). Besides, it has low brain–blood barrier (BBB) permeability, which means that is not effective for the treatment of late-stage HAT.

Suramin reached the market in 1920 and is still one of the treatment options for the first stage of rHAT. Its most frequent side effect is urticarial rash (which affects around 90% of the patients). Other adverse events include reversible nephrotoxicity, pyrexia, and nausea (Nagle et al., 2014).

For the treatment of second stage of HAT, an arsenic derivate has been recommended for years: the melarsoprol. It was employed from 1949 (Nok, 2003), but its toxicity is extremely high, causing severe encephalopathic syndrome in some cases, which is associated to high mortality rate (Kennedy, 2004).

Another approved drug for the treatment of HAT is eflornithine, a repurposed drug first explored as anticancer agent (Nwaka and Hudson, 2006). Eflornithine has been used as an alternative to melarsoprol, and its mode of action involves the inhibition of the enzyme ornithine decarboxylase (O'Shea et al., 2016). Its side effects are less severe than those of melarsoprol, but it requires intravenous administration because of its low oral bioavailability. This is an important drawback if one considers that most patients with HAT have limited access to adequate health facilities, complicating the follow-up. Furthermore, it is active only against *T. b. gambiense*. The outcome is significantly improved when eflornithine is combined with nifurtimox. As monotherapy in *T. b. gambiense* infections, nifurtimox is effective against both the early and late stages, but it has a very variable cure rate (30–80%) and high toxicity upon long-term administration (Bouteille et al., 2003). Nifurtimox combined with eflornithine (Nagle et al., 2014), though, is an interesting therapeutic choice, and it was incorporated to the WHO List of Essential Medicines for the treatment of gHAT (World Health Organization, 2020a).

In 1983, Raether and Seidenath reported fexinidazole as a highly active new antiparasitic drug effective against trichomonads, *Entamoeba histolytica* and *T. cruzi*, but the project was abandoned by the pharmaceutical company Hoechst when its tropical disease program was shut down (Raether and Seidenath, 1983). Thanks to the non-profit Drugs for Neglected Diseases initiative (DNDi) organization and the pharmaceutical company Sanofi joint efforts, this drug finalized preclinical stage and underwent clinical trials (Fairlamb, 2019). It has been approved as the first all oral medication against both hemolymphatic and meningoenchepalitic gHAT, and it is undergoing a 5-year clinical trial to prove its efficacy against rHAT (ClinicalTrials.gov NCT03974178, 2020; Drugs for Neglected Diseases initiative, 2020e). After oral administration, fexinidazole is readily distributed throughout the body, including the brain (Tarral et al., 2014).

Currently, the oxaborole SCYX-7158 is undergoing clinical trials for the treatment of HAT. Encouraging results of SCYX-7158 in animal models made the responsible researchers suspect that it might be a good treatment option for late-stage HAT (Jacobs et al., 2011). Two other oxaborole compounds, SCYX-1608210 and SCYX-1330682, have shown good performance in animal models of the disease (Drugs for Neglected Diseases initiative, 2020c). In 2015, a placebo-controlled, randomized, double-blind study was completed, which assessed the tolerability and pharmacokinetic parameters of SCYX-7158 (ClinicalTrials.gov NCT01533961, 2020). The study confirmed that the drug readily crosses the BBB, thus being a promising candidate to treat late-stage HAT, although some adverse effects such as gastrointestinal reactions and headaches were observed (Drugs for Neglected Diseases initiative, 2020c). Based on these results, a phase II/III trial started in 2017, to evaluate the effectiveness and safety of SCYX-7158 as an oral treatment for adult patients with gHAT (ClinicalTrials.gov NCT03087955, 2020). The chosen dosage regimen involved a single administration of 960 mg. Phase II/III results are awaited in the next years (Dickie et al., 2020).

Table 1 summarizes the therapeutic scenario for HAT.

**Table 1:** Drugs approved or under clinical trials for the treatment of human African trypanosomiasis (HAT).

**Drugs**	**Stage of the disease**	**Administration via**	**Adverse reactions**	**References**
**WHO-recommended drug treatments**				
Pentamidine				
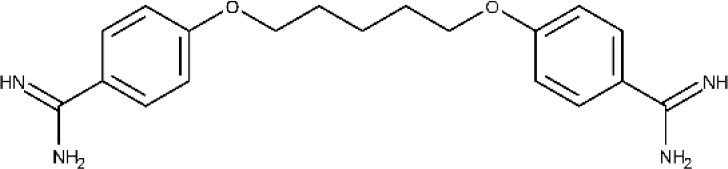	First stage	Intramuscular	Hyperglycemia or hypoglycemia, prolongation of the QT interval on electrocardiogram, hypotension, and gastrointestinal features	(Nok, 2003)
Suramin				
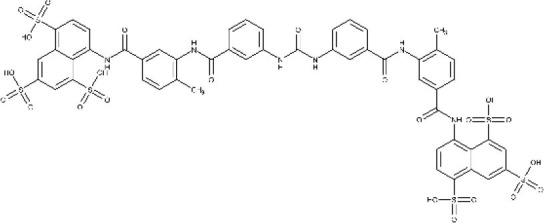	First stage	Intravenous	Renal failure, skin lesions, anaphylactic shock, bone marrow toxicity, and neurological complications such as peripheral neuropathy	(Nok, 2003)
Melarsoprol				
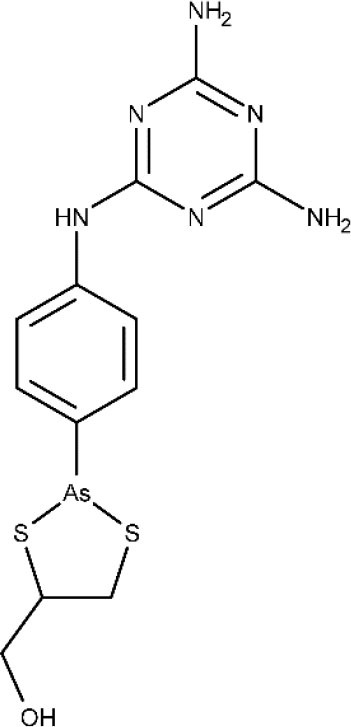	Second Stage	Intravenous	Reactive arsenical encephalopathy (RAE) has been attributed to the toxic effect of melarsoprol, peripheral neuropathy, cutaneous reactions, renal or hepatic dysfunction, allergic or hypersensitivity reactions	(Nok, 2003; Eperon et al., 2014)
Eflornithine				
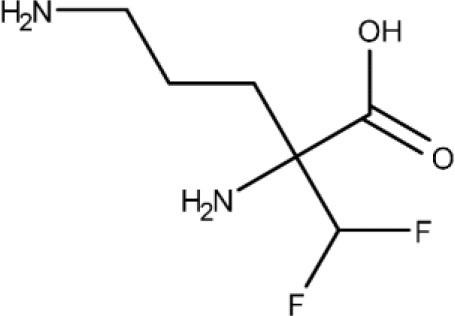	Second stage. Only useful against *T. b. gambiense*	Intravenous	Generally, are reversible after the end of treatment. Convulsions, gastrointestinal symptoms such as nausea, vomiting, and diarrhea; bone marrow toxicity leading to anemia, leukopenia, and thrombocytopenia	(Burri, 2010; Alirol et al., 2013)
Nifurtimox & Eflornithine	First and second stage	Intravenous (eflornithine) Oral (nifurtimox)	Convulsions, gastrointestinal symptoms such as nausea, vomiting, and diarrhea; Genotoxicity, neurotoxicity	(Burri, 2010; Yun et al., 2010; Kuemmerle et al., 2020)
Fexinidazole				
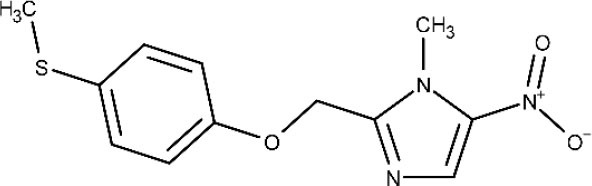	First and second stage of *T. b. gambiense* infection	Oral	Headache and vomiting	(Tarral et al., 2014; Fairlamb, 2019; ClinicalTrials.gov NCT01685827, 2020; ClinicalTrials.gov NCT03025789, 2020)
**Undergoing clinical trials**				
Acoziborole (SCYX-7158)				
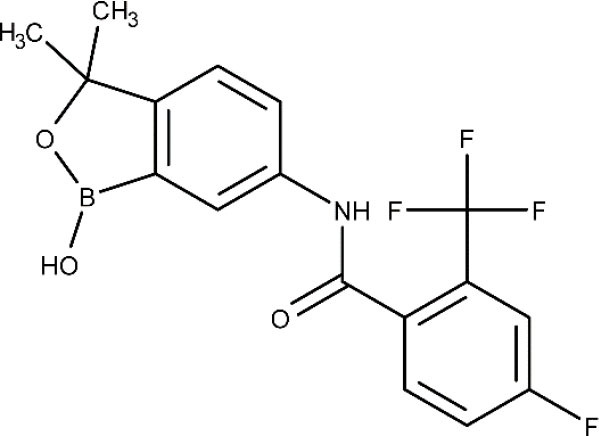	First stage Second stage	Oral	Diarrhea, constipation, nausea, vomiting, abdominal pain, headaches	(ClinicalTrials.gov NCT01533961, 2020; ClinicalTrials.gov NCT03087955, 2020; Dickie et al., 2020)
Fexinidazole				
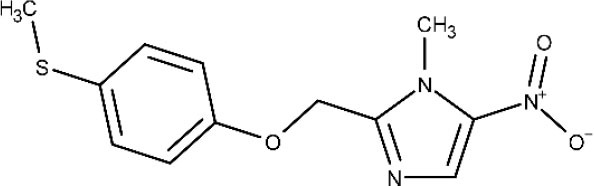	First and second stage of *T. b. rhodesiense* infection	Oral	Results have not been disclosed yet	(ClinicalTrials.gov NCT03974178, 2020)

### Lipid Nanosystems Encapsulating Approved Drugs

Limited permeability across the blood–brain barrier by therapeutic agents is one of the main limitations that an efficacious trypanocidal agent may present to be considered a valid option to treat late-stage HAT. For instance, pentamidine is mostly ineffective once the parasite has invaded the CNS, and such lack of efficacy is thought to respond to its inability to enter the brain. The limited bioavailability of pentamidine in the brain has been demonstrated in mice by Sanderson et al. (2009), whose results suggested that the drug distribution to the brain is conditioned by active efflux mediated by P-glycoprotein and multidrug resistance–associated proteins. Different approaches have been used for the delivery of drugs to the brain, such as intracerebroventricular administration or intranasal delivery. Among them, vector-mediated brain delivery, involving enhanced brain bioavailability through drug-carrier conjugates, seems particularly promising because of its versatility and reduced side effects compared to other delivery options (Li et al., 2017; Gondim et al., 2019). Omarch et al. (2019) investigated the permeability of polycaprolactone nanoparticles and liposomes containing both pentamidine across a monolayer of immortalized mouse brain endothelioma cells. Pentamidine-loaded polycaprolactone nanoparticles showed a mean diameter of 267.6 nm and zeta potential of −28.1 mV, whereas liposomes had a mean diameter of 119.6 nm and zeta potential of 11.78. Both systems displayed low dispersity and similar loading capacity. After 24 h, liposomes and polycaprolactone nanoparticles transported 87 and 66% of the doses, respectively. Besides, free pentamidine penetration was only 63% of the dose. The data suggested that lipid structures can be promising nanocarriers to increase brain bioavailability of pentamidine.

### Lipid Nanosystems Encapsulating Non-approved Drugs

Like pentamidine, diminazene aceturate is a trypanocidal aromatic diamidine of veterinary use. Its mode of action involves the irreversible inhibition of S-adenosyl-l-methionine decarboxylase of the trypanosome (Pépin and Milord, 1994). In the past, diminazene aceturate has been used off-label by national control programs and clinicians in many HAT endemic regions of the world, with the consequent ethical controversy (Pépin and Milord, 1994). Diminazene presents limited stability and brain bioavailability (Kroubi et al., 2010).

Kroubi et al. (2010) obtained cationic polysaccharide nanoparticles with a lipid core (anionic phospholipids). It was previously observed that these types of nanocarrier could be endocytosed by the blood–brain barrier endothelial cells and did not activate the complement system, which strongly suggests a stealth behavior (Jallouli et al., 2007; Paillard et al., 2010). The authors tested two drug loading approaches, in process (at 80°C) and post-loading (at room temperature), although poor stability was observed in the former approach. The hydrodynamic diameter of the prepared nanoparticles was 74 nm, with low dispersion and a zeta potential +29 mV. Possibly because of the porous nature of the nanosystem, the mean size was not changed by the drug load. The loading capacity and stability of the hybrid nanoparticles greatly depended on the drug to phospholipid ratio: formulations with a drug-to-phospholipid ratio <5% were stable in terms of size, charge, and drug loading for at least 6 months (4°C). It was also observed that the encapsulation of the drug protected it against oxidation, being stable for at least 6 months at 4°C. Furthermore, *in vitro* assays on *T. brucei* showed an increased efficacy of the loaded nanoparticles compared with the free drug.

## Chagas Disease (American Trypanosomiasis)

Chagas disease, caused by the protozoan *T. cruzi*, is endemic to Latin America, but it has also spread to non-endemic countries because of human migration. It is transmitted mainly through an insect vector commonly known as “kissing bug” or *vinchuca* (*Triatoma infestans*), but other transmission ways have become increasingly relevant, including congenital transmission, blood transfusion, and organ transplant (Pereira and Navarro, 2013; World Health Organization, 2020b). It presents itself in two or three stages (acute and chronic, or acute, latent/undetermined, and chronic), depending on bibliographic sources. The acute stage is associated with a high parasite load in blood and displays absent or mild and unspecific symptoms. In the chronic stage, the parasite predominates in other tissues (mainly in the heart and digestive muscles), with up to 30% of patients experiencing cardiac disease and around 10% displaying digestive complications. The chronic stage often leads to sudden death due to cardiac cardiomyopathy (World Health Organization, 2020b).

### Chagas Disease Treatment

Only two trypanocidal drugs are available to treat Chagas disease: benznidazole and nifurtimox, with both being discovered around 1970 (World Health Organization, 2020a). The mode of action of benznidazole involves the covalent modification of biomolecules, due to the generation of reactive intermediates emerging from reduction of the nitro group (Mecca et al., 2008). It is highly efficacious in the acute stage; however, as the disease progresses, the efficacy decreases, and the cure rate in the chronic phase is estimated around 10–20% (Prata, 2001). The BENEFIT was a multicenter, prospective, randomized study including patients with Chagas' cardiomyopathy who received benznidazole or placebo for up to an 80-day period (Morillo et al., 2015). Two thousand eight hundred fifty-four patients were followed up for more than 5 years after the intervention. Sixty-six percent of the patients treated with benznidazole reverted positive polymerase chain reaction (PCR) results in comparison to 33.5% of patients in the placebo arm. This positive outcome, nonetheless, was overshadowed by the fact that cardiac deterioration was not prevented in the active arm. It is worth mentioning that benznidazole is often poorly tolerated, presenting adverse reactions/side effects such as rashes, peripheral neuropathy, hypersensitivity syndromes with fever, lymphadenopathy, exfoliative dermatitis, anorexia, nausea, vomiting, and insomnia (World Health Organization, 2020a), which often lead to treatment interruption.

Nifurtimox, the second-line treatment, is prescribed in cases where benznidazole is not well-tolerated. Its mode of action, again, relates to the reduction of the nitro group, leading to the formation of reactive oxygen species (Urbina and Docampo, 2003, Maya et al., 2007). As previously discussed, nifurtimox is also associated to several complications, including anorexia, psychic disorders, irritability, insomnia, nausea, and diarrhea (Bern et al., 2007).

Regarding recent and ongoing clinical trials, disappointing results were obtained with the repurposed antifungal posaconazole. Docampo et al. (1981) were the first to suggest the use of azole compounds against *T. cruzi*. In 2010, posaconazole was included in a clinical trial to assess its efficacy against chronic Chagas disease. It did show trypanosomal activity, but more posaconazole patients (compared with benznidazole treatment) showed failure throughout the follow-up of the treatment (Molina et al., 2014; ClinicalTrials.gov NCT01162967, 2020). Later, a second trial was started to study the efficacy of oral posaconazole for the treatment of asymptomatic Chagas disease (ClinicalTrials.gov NCT01377480, 2020), but once again, the investigators found better performance in the control arm (Morillo et al., 2017). Highly sensitive bioluminescence studies were later performed with bioluminescent *T. cruzi* to assess parasite survival in mice, after treatment with either posaconazole or benznidazole; cyclophosphamide-induced immunosuppression was used to facilitate the detection of relapse (Fortes Francisco et al., 2015). While 20-day treatment with benznidazole was successful to achieve sterile cure, posaconazole failed in almost all cases. In the acute phase, the adipose tissue appears to be the major reservoir of recurrence in mice under posaconazole therapy. Inadequate choice of the dose at clinical trials and emergence of azole-resistant parasites should also be considered as possible explanations to the failure at the clinical studies (Campos et al., 2017; Villalta and Rachakonda, 2019).

E-1224 (a prodrug of ravuconazole) is another azole being investigated as potential therapy for Chagas (Diniz et al., 2018). In 2011, a proof-of-concept study was started in individuals with chronic indeterminate Chagas disease (ClinicalTrials.gov NCT01489228, 2020) but the outcome did not cover the expectations, as the control group showed better results after 1-year treatment (Torrico et al., 2018). More recent studies are focusing on the evaluation of improved treatment regimens of benznidazole in monotherapy and in combination with E1224 (ClinicalTrials.gov NCT03378661, 2020).

After Bahia et al. (2012) demonstrated the *in vitro* an *in vivo* effects over *T cruzi*, fexinidazole entered a phase II trial to determine its efficacy in adults with chronic indeterminate disease, finding that the drug was highly effective at the lowest dose (ClinicalTrials.gov NCT02498782, 2020; Drugs for Neglected Diseases initiative, 2020j). A second study started a few years later in which 3, 7, and 10 days of treatment with low doses of the drugs are being evaluated, to establish the minimal effective and safe dose to treat adult patients undergoing chronic indeterminate Chagas disease (ClinicalTrials.gov NCT03587766, 2020). Results are expected in 2020.

A second study started a few years later for adult patients' therapy having chronic indeterminate Chagas sickness for which they received 3, 7, and 10 days of treatment with low drug doses.

As cardiac deterioration is the major complication in chronic Chagas disease, there are also some undergoing trials focused on ameliorating Chagas cardiomyopathy. In 2009, a clinical trial started to estimate the effect of selenium treatment on prevention of heart disease progression in cardiac patients with Chagas (ClinicalTrials.gov NCT00875173, 2020). Selenium has prevented myocardial lesions in acute and chronic models (Souza et al., 2010). In 2018, there were protocol modifications (Holanda et al., 2018), and recruitment of patients has been reestablished. Results should be expected soon, and if the hypothesis of the trial is confirmed, the inclusion of this micronutrient in the daily diet could have a therapeutic effect on Chagas myocardiopathy.

Another example of a repurposed drug that could be used to treat the Chagas-associated cardiomyopathy is the antigout agent colchicine, which has demonstrated cardioprotective effects (reduced fibrosis and diminished inflammation in the cardiac tissue) (Fernandes et al., 2012). Positive effects on myocardial remodeling, linked to interference in the synthesis of collagen, were also reported. Currently, a clinical study is in the recruiting phase, with the first results being expected by 2021 (ClinicalTrials.gov NCT03704181, 2020).

Finally, amiodarone is a class III antiarrhythmic agent and was first found as an antimycotic (Courchesne, 2002; Hejchman et al., 2012). Later, Benaim et al. (2006) reported its trypanocidal effects against *T. cruzi* (Bellera et al., 2013). The potential benefits on both its cardiovascular activity and the intracellular Ca^2+^ regulation of the parasite make this compound particularly attractive. An ongoing phase III clinical trial (ATTACH) was designed to test the effect of amiodarone, administered over 6 months, in subjects with mild to moderate Chagas cardiomyopathy; secondarily, potential trypanocidal effects associated with beneficial clinical effects will be explored (ClinicalTrials.gov NCT03193749, 2020).

Table 2 presents a summary of the therapeutic scenario for Chagas disease, including approved drugs and drugs that have or are undergoing clinical trials.

**Table 2:** Summary of the therapeutic scenario for Chagas disease, including WHO-recommended therapies, and recent/undergoing clinical trials.

**Drugs**	**Stage of the disease**	**Administration via**	**Adverse reactions**	**References**
**Drugs used to treat Chagas disease recommended by WHO**				
Benznidazole First line of treatment 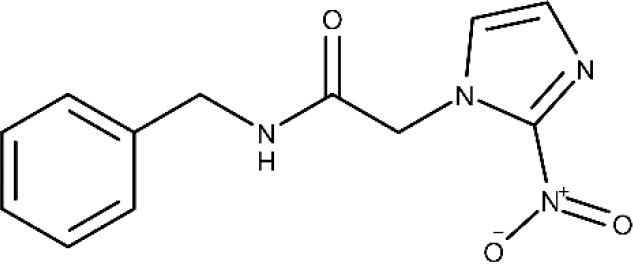	Acute phase Chronic phase	Oral	Rashes, peripheral neuropathy, hypersensitivity syndromes with fever, lymphadenopathy, exfoliative dermatitis, anorexia, nausea, vomiting, weight loss, and insomnia	(Bern et al., 2007; Mecca et al., 2008; Crespillo-Andújar et al., 2020; ClinicalTrials.gov NCT03191162, 2020; ClinicalTrials.gov NCT03981523, 2020)
Nifurtimox 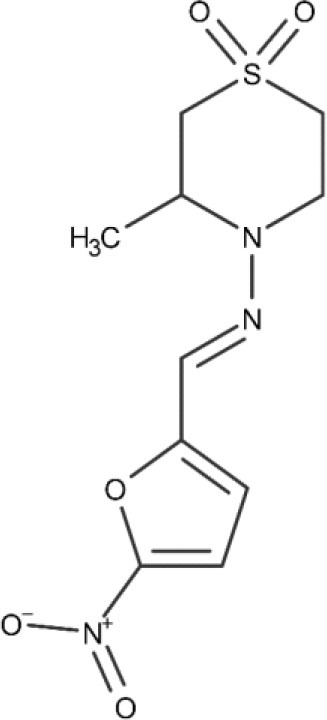	Acute phase (for those patients who do not tolerate benznidazole)	Oral	Anorexia, weight loss, psychic disorders, irritability, insomnia, nausea, diarrhea	(Urbina and Docampo, 2003; Bern et al., 2007; Maya et al., 2007; Boiani et al., 2010; Hall et al., 2011; ClinicalTrials.gov NCT03981523, 2020)
**Undergoing clinical trials**				
Posaconazole 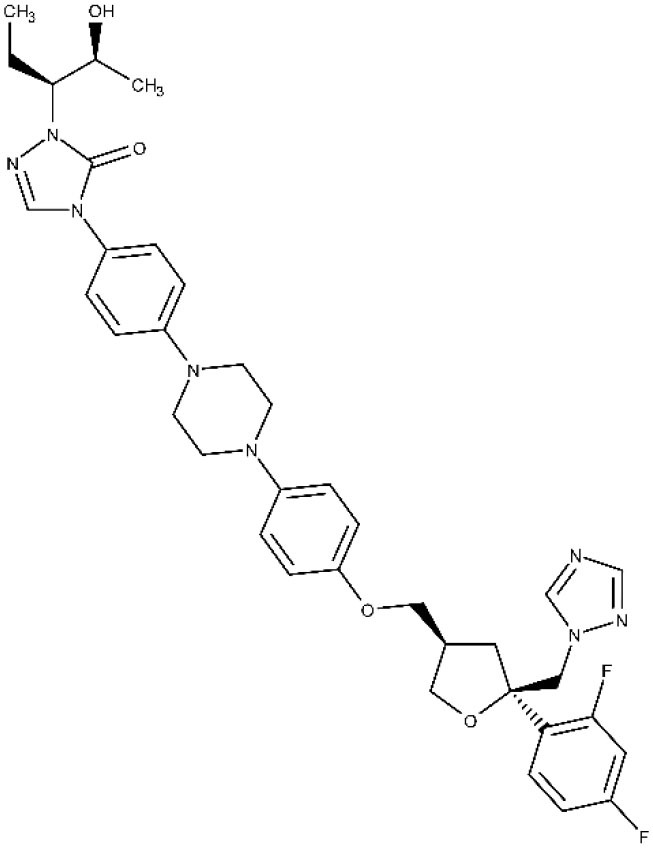	Acute phase Chronic phase	Oral	Drug interactions related to CYP3A4 inhibition. Caution must be taken when coadministered with other CYP3A4 substrates. Care must be taken when administered to a patient with arrhythmic disorders or taking proarrhythmic drugs	(Molina et al., 2014; Morillo et al., 2017; Urbina, 2017; ClinicalTrials.gov NCT01377480, 2020; Echeverría et al., 2020)
Fexinidazole 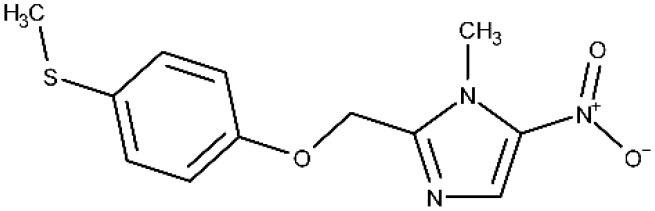	Acute phase Chronic phase	Oral	Headache and vomiting of acceptable intensity	(Neal and van Bueren, 1988; Bustamante and Tarleton, 2014; ClinicalTrials.gov NCT02498782, 2020; ClinicalTrials.gov NCT03587766, 2020)
Ravuconazole and E-1224 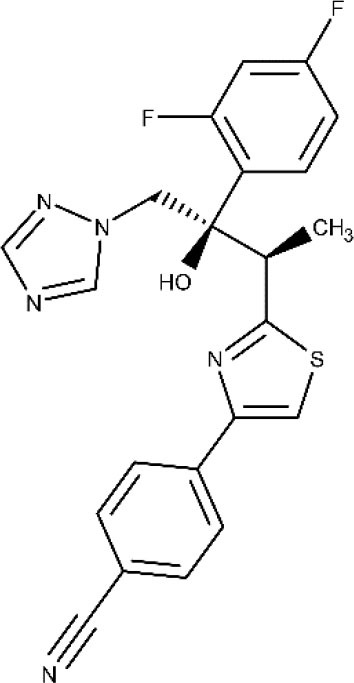	Acute phase	Oral	Not informed	(Urbina et al., 2003; ClinicalTrials.gov NCT01489228, 2020; ClinicalTrials.gov NCT03378661, 2020; ClinicalTrials.gov NCT03892213, 2020)
*Ravuconazole E-1224* 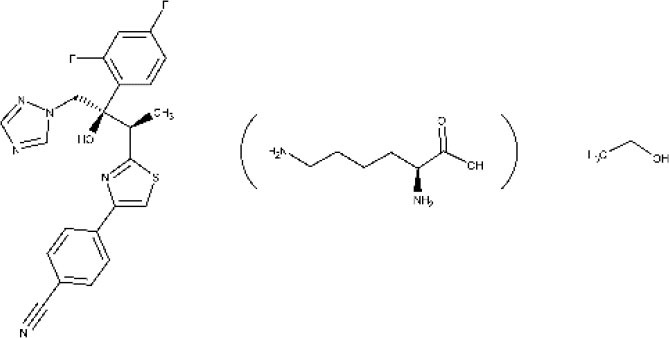	Chronic phase			
Selenium	For prevent Chagas cardiomyopathy	Oral	Not reported	(Holanda et al., 2018; ClinicalTrials.gov NCT00875173, 2020)
Colchicine 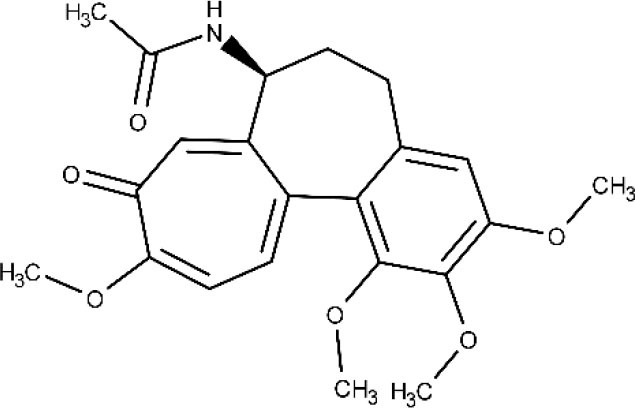	For prevent Chagas cardiomyopathy	Oral	Not reported	(ClinicalTrials.gov NCT03704181, 2020)
Amiodarone 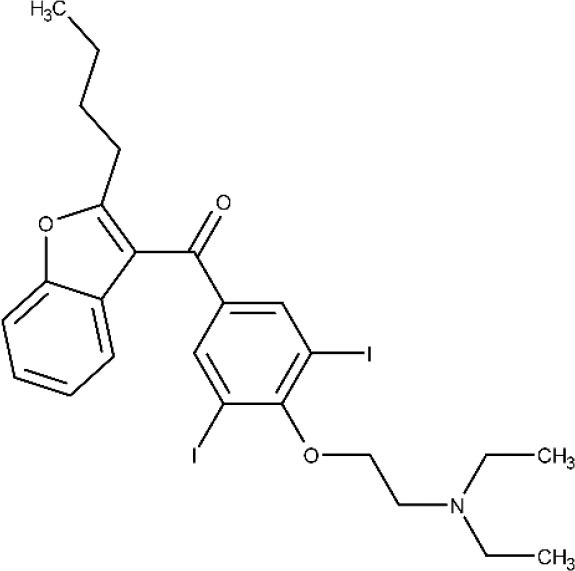		Oral	Not reported	(Bellera et al., 2013; Carmo et al., 2015; ClinicalTrials.gov NCT03193749, 2020)

### Lipid Nanosystems Encapsulating Approved Drugs

Although much of the efforts toward encapsulation of benznidazole and nifurtimox has focused on polymeric nanosystems, a considerable number of studies have also been published reporting the use of lipid-based nanocarriers, especially in the case of the first-line therapy benznidazole (Arrúa et al., 2019).

Morilla et al. (2002) reported the obtention of benznidazole multilamellar liposomal formulations; they proposed that such strategy would compensate benznidazole low solubility and improve its biodistribution. Among many tested formulations, the highest drug load was observed in hydrogenated phosphatidylcholine from soybean: cholesterol: distearoyl-phosphatidylglycerol in a molar ratio of 2:2:1. Drug loading of 2 g of the drug per 100 g of lipids was achieved. A 450-fold dilution in buffer at 37°C led to a reduction in the quantity of drug associated to liposomes from 2 g to 0.25 g/100 g of total lipids at 65% of drug lost per minute since the first minute and by severe decrease of drug release (0.4% of drug lost per minute) in the next hour. The low efficiency of the drug carrier concomitantly with high amount of drug loss can be attributed to the liposomal thermodynamic instability under physiological conditions (Cacicedo et al., 2016). Subsequently, the same authors investigated the ability of such liposomes to enhance the delivery of benznidazole to the liver in rats (Morilla et al., 2004). Three-fold raise in benznidazole was accumulated in the liver, together with 1.1 μg/mL BNZ in blood, which is 30% lower than the blood BNZ concentration achieved upon intravenous administration of free drug happened 4 h post-ijnjection. Besides, the increase of BNZ liver uptake had no effect on parasitemia levels of mice infected with the RA *T. cruzi* strain.

A diversity of nanocarriers encapsulating benznidazole (among them, several lipid-based systems) were studied by the BERENICE consortium (BEnznidazol and triazol REsearch group for Nanomedicine and Innovation on Chagas disease, a project originally conceived to develop low-cost therapeutic interventions for Chagas disease using nanotechnology to reduce the final dose of the drug) (Vinuesa et al., 2017). The drug delivery systems tested included different cyclodextrins, quatsomes, liposomes, SLN, and NLC formulations. Liposomal formulations sedimented in time, although this behavior was prevented through PEGylation. The release profiles of the SLN and NLC showed a considerable initial burst release. In the case of SLN, those with the higher benznidazole load (20%) released 18% of the load at 1.5 h. Subsequently, the release rate was much slower (the maximum drug release was 19.5%). The maximum release achieved with the NLC was higher than that of SLN (up to 55% of the load for the highest drug load, i.e., 30%), and the initial burst was reached only 5 h later. Regarding cytotoxicity of the empty carriers, quatsomes resulted to be highly cytotoxic at high concentrations. Liposomal preparations were much less cytotoxic, with some of them displaying negligible toxicity. NLC showed dose-dependent (but low) cytotoxicity: empty NLC showed at most 10% toxicity (at 100 μg/mL) on mice fibroblasts. A paradoxical (unexplained) behavior was registered, since the higher the benznidazole load, the smaller the toxicity detected. Similarly, CD had low cytotoxicity. None of the formulations tested provided substantial improvement on the trypanocidal effect of benznidazole, and only cyclodextrin complexes achieved some improvement in the selectivity index. These discouraging results possibly led the BERENICE team to discard the nanotechnology approach.

Another recent study investigated the ability of oil-in-water nanoemulsions to modify benznidazole release and their impact on the parasite viability (Streck et al., 2019). Dispersions of medium-chain triglycerides were stabilized by using phosphatidylcholine and sodium oleate. The nanoemulsion increased benznidazole apparent aqueous solubility and led to a slower drug release. Cell viability studies revealed that the nanoformulation enhanced the cytotoxicity at high concentrations (200 μg mL^−1^). The tested formulations also induced a remarkable increment of efficacy against epimastigotes and trypomastigotes, in comparison with the free drug.

### Lipid Nanosystems Encapsulating Non-approved Drugs

In 1999, the performance of three marketed lipid amphotericin B formulations (i.e., the liposomal AmBisome™, the lipid complex Abelcett™, and Amphocil™, a colloidal dispersion) was challenged *in vitro* and *in vivo* against amphotericin B deoxycholate (Fungizone™). Amphocil and Fungizone showed 42- and 7-fold more activity than Abelcet and AmBisome against amastigotes of *T. cruzi* in macrophages, respectively. However, the tested formulations showed similar performance against *T. cruzi* amastigotes in cultures of Vero cells. Interestingly, administration of a single 25 mg/kg dose of AmBisome inhibited acute infections of *T. cruzi* in mice, whereas at the same dose the other lipid formulations enhanced the survival rate, but the infections were not eliminated in all animals (Yardley and Croft, 1999). These results clearly evidence the absence of *in vitro*–*in vivo* correlation. In good agreement with these findings, Cencig et al. (2011) proved that the increase in survival and parasitemia decreases in the course of acute or chronic phases of *T. cruzi* of infected mice by six intraperitoneal AmBisome injections. Analysis by quantitative PCR of infected mice showed significant parasite load reductions in heart, spleen, skeletal muscle, liver, and adipose tissues in both phases. Noteworthy, earlier administration of the amphotericin B formulation led to increased efficacy in parasite load decreases in spleen and liver, and recurrent drug administration also had beneficial effects in the parasite load in heart and liver during the chronic phase. Unfortunately, immunosuppression with cyclophosphamide boosted the infection to parasite levels equivalent to untreated animals acutely infected. These results strongly suggest that, at least in the assayed dosing schedule, the liposomal formulation failed to fully cure the infection.

Morilla et al. (2005) encapsulated the trypanocidal drug etanidazole in pH-sensitive liposomes made of dioleoyl-phosphatidylethanolamine and cholesteryl hemisuccinate 6:4. The liposomes were also loaded with a fluorescent probe. Their mean diameter was around 380 nm. The mean size drastically changed (5-fold increase) when the pH of the external media dropped from 8.7 to 3 (it would have been interesting, though, to study the behavior at intermediate, physiologically relevant pH values). It was demonstrated that they were phagocytosed by uninfected and *T. cruzi*–infected macrophages, eliciting an appreciable trypanocidal effect, while control with free drug did not show any therapeutic effect. Intravenous administration of the encapsulated drug to infected mice also decreased parasitemia levels, whereas administration of a 180-fold higher dose of the free drug had no positive effect.

Carneiro et al. (2014) developed SLN containing the potential trypanocidal drug lead 5-hydroxy-3-methyl-5-phenyl-pyrazoline-1-(S-benzyldithiocarbazate). The mean diameter of the loaded SLN was 127 nm with low dispersity; the zeta potential revealed a considerably negative surface charge (−56.1 mV). A high entrapment efficiency was also demonstrated. The *in vitro* and *in vivo* performances of the encapsulated drug, the free drug, and benznidazole were compared. The SLN system outperformed the other treatments in a mice model of infection, both in terms of parasitemia reduction. Liver inflammation and damage were also diminished by the drug-loaded nanocarrier.

More recently, Spósito et al. (2017) resorted to a self-emulsifying formulation to efficiently deliver ravuconazole, a low-solubility drug pertaining to class II of the Biopharmaceutical Classification System. The emulsifying system considerably enhanced the *in vitro* drug dissolution extent and rate in comparison with the free drug (20 vs. 3% at 6 h). The formulation clearly improved the *in vitro* activity of the drug against the intracellular stage of *T. cruzi*. Cruz-Bustos et al. (2012) used *Quillaja* saponin (an immunostimulant agent used as vaccine adjuvant, which forms nanometer pentagonal dodecahedral balls known as immunostimulant complexes) in the design of targeted nanocapsules loaded with actinomycin D and functionalized with anti–*T. cruzi* antibodies. Confronted with *T. cruzi* epimastigotes, the encapsulated drug elicited trypanocidal effects in a dose-dependent manner, at much lower concentrations than the free drug control. Remarkably, this is to our best knowledge the first reported targeted lipid nanocarrier against Chagas disease.

De Morais et al. (2019) reported the obtention of polymeric micelles and phospholipid 2-dipalmitoyl-sn-glycero-3-phosphocholine liposomes containing the photosensitizer drug hypericin. The mean size, polydispersity index, or zeta potential were not informed. Confronted with *T. cruzi* trypomastigotes, pluronic micelles showed efficacious even in the absence of light, with their EC_50_ around 7 μmol L^−1^. Under light, the best result was achieved by the liposomal system, with EC_50_ around 0.31 μmol L^−1^. Although free hypericin showed a very similar potency, authors underlined that the encapsulated drug would be protected against blood components, which constitutes and additional advantage of the loaded liposomes. At last, Parra et al. (2020) reported the obtention of double targeted imiquimod-containing nanovesicles prepared from lipids from the archaebacterium *Halorubrum tebenquichense*, to induce protection against *T. cruzi* infection. The therapeutic efficacy of the vesicles was assessed in a mice model of acute infection, and it was shown that it prevented mortality, reduced parasitemia levels (although not as much as benznidazole), and reduced myocardial and skeletal muscle damage (even more than benznidazole).

Violacein, a natural colorant produced by some Gram-negative bacteria, showed a predominantly apoptotic effect in developmental forms of *T. cruzi* (Y strain) and selectivity index of 9 (Canuto et al., 2019). However, the poor aqueous solubility of violacein is a serious obstacle for development pharmaceutical formulations. In the present year, Rivero Berti et al. (2020) developed a novel SLN formulation of violacein containing surface-active ionic liquids (SAILs) of the cation 1-alkylimidazolium and a fluorescent tracer DiOC_18_. The results indicate 6-fold incorporation of the SLN in mammalian cell cultures with high apoptotic activity (Rivero Berti et al., 2020).

## Leishmaniasis

Leishmaniasis is caused by more than 20 different species of the *Leishmania* genre, and it is spread to mammalians through the bite of female phlebotomine infected sandflies. The disease presents itself in three forms (mainly): cutaneous (the most common), visceral (the most severe form, also known as kala-azar), and mucocutaneous. The epidemiology of leishmaniasis involves many aspects such as type of sandfly species, parasite, ecological features of the transmission places, and human behavior (World Health Organization, 2020c,d).

### Leishmaniasis Treatment

Alike the already described HAT treatment scenario, there is a wide range of treatments that may or may not be applicable, depending on the cost, stage of the disease, parasite species, geographic location, and tolerability. It is difficult to develop a single drug capable of universally treating the disease, because of the huge variability of strains and clinical manifestations. According to the report of a meeting of the WHO Expert Committee on the Control of Leishmaniases, the recommended drugs are amphotericin B (traditional and liposomal formulation), pentavalent antimonial, paromomycin, miltefosine, and pentamidine.

Amphotericin B (AmBD) is an aminoglycoside with biostatic or biocidal properties that binds to sterols (ergosterol) in the cell membrane of microorganisms, thus creating a transmembrane channel and disrupting the membrane integrity (Wortmann et al., 2010). Its disadvantages include cost, the route of administration (slow intravenous infusion), and systemic and renal toxicity (Stone et al., 2016). In 1997, a liposomal formulation of AmBD—LAMBD—was authorized by the Food and Drug Administration (FDA). LAMBD diminishes the incidence of the severe side effects, thus improving tolerability. However, the production cost of the liposomal formulation is still a key barrier to accessibility in endemic countries.

Pentavalent antimonials, mainly meglumine antimoniate and sodium stibogluconate, are the first-line drugs to treat leishmaniasis (Frézard et al., 2009; Miranda-Verastegui et al., 2009). They have been the treatment of choice since 1940. It has been proposed that sodium stibogluconate may act as a prodrug that is later reduced *in vivo*, disrupting the cell thiol redox potential. It was also shown that these compounds bind to DNA I topoisomerase inhibiting plasmid DNA unwinding. Among their many side effects are nausea, vomiting, skin rashes, abdominal colic, and cardiotoxicity (Frézard et al., 2009). After all those years as the main clinical choice, the alarming growing rate of antimonial resistance is not surprising (Arevalo et al., 2001).

Discovered in 1950, paromomycin is an aminoglycoside antibiotic originally isolated from *Streptomyces rimosus* (Wiwanitkit, 2012). It binds to 16S rRNA and consequently inhibits protein synthesis by increasing the error rate in the translation process. It also disrupts the pathogen membrane fluidity and lipid metabolism. In 2006, paromomycin was approved by the FDA as an antileishmanial medication against the visceral presentation of the disease. Although it produces several undesirable side effects such as abdominal cramps and diarrhea (Wiwanitkit, 2012), it is still considered as one of the most cost-effective treatments. It is still considered as one of the most cost-effective treatments. The combination of puromycin with sodium stibogluconate (SSG&PM) has been demonstrated safe and effective, with the advantages of being a shorter and less expensive treatment. SSG&PM has been recommended by WHO as first-line treatment for visceral leishmaniasis (Kimutai et al., 2017; Drugs for Neglected Diseases initiative, 2020i).

The alkyl phospholipid miltefosine was the first oral drug registered to treat visceral leishmaniasis. Its mechanism of actions is not still fully understood but it is related to programmed cell death triggered by alkyl phospholipids. Miltefosine was first considered as potential treatment in breast cancer and other solid tumors, but it was discontinued after signs of severe gastrointestinal toxicity (Sundar and Olliaro, 2007). Later, it demonstrated high efficacy against *Leishmania* both *in vitro* and *in vivo* (Croft et al., 1987). In 2014, it was approved as the first oral treatment of leishmaniasis by the FDA. Its disadvantages include the already mentioned gastrointestinal toxicity, hand in hand with high cost and teratogenicity potential (Soto and Soto, 2006). It did not take long to show the synergy effects *in vitro* between miltefosine and liposomal amphotericin B (Seifert and Croft, 2006). Moreover, a retrospective study demonstrated a good cure rate (>80%) in human immunodeficiency virus patients coinfected with visceral leishmaniasis treated with that combination (Abongomera et al., 2018; Drugs for Neglected Diseases initiative, 2020a). Currently in the recruiting phase (phase III), this is a randomized study to test the effectiveness of oral miltefosine in combination with intravenous liposomal amphotericin and intramuscular paromomycin in patients with post Kala Azar dermal leishmaniasis (PKDL) (ClinicalTrials.gov NCT03399955, 2020; Drugs for Neglected Diseases initiative, 2020g,h). The combination of miltefosine with paromomycin was found successful over the intracellular amastigote stage and in diminishing parasite loads in the liver, spleen, and bone marrow of an *in vivo* model (Hendrickx et al., 2017). In 2017, a randomized trial (phase III) was developed to compare the effect of miltefosine in combination with paromomycin for the treatment of visceral leishmaniasis in adults and children (ClinicalTrials.gov NCT03129646, 2020; Drugs for Neglected Diseases initiative, 2020g). Results of the study are expected by the end of 2020.

Pentamidine isethionate has already been discussed for the treatment of HAT. Pentamidine is a broad-spectrum anti-infective agent active against several parasitic worms, protozoa, and fungi with relatively toxic effects, thus requiring careful monitoring during therapy (Hafiz and Kyriakopoulos Pentamidine, 2020; World Health Organization, 2020d). It has been used as monotherapy or in combination to treat cutaneous and visceral leishmaniasis. However, the adverse effects of the drug are severe, and consequently, it is preferably averted.

There are some new chemical entities that are undergoing clinical studies (phase I). DNDI-0690 is a nitroimidazole that displays very promising (*in vitro*) activity against laboratory strains of *Leishmania infantum* and *in vivo* in the early curative hamster model (Van den Kerkhof et al., 2018). Furthermore, based on *in vivo* bioluminescence imaging, only two administrations of this compound were enough to lower the *Leishmania mexicana* parasite load by 100-fold in a murine model of cutaneous leishmaniasis (Wijnant et al., 2019). In 2015, DNDi-0690 was nominated as a preclinical candidate, intended to be used as oral treatment for visceral and cutaneous leishmaniasis. Last year, the first trial in humans was started to evaluate the safety and tolerability of a single administration of this compound (ClinicalTrials.gov NCT03929016, 2020).

Other compound under phase I is the DNDI-6148, from the oxaborole class (Drugs for Neglected Diseases initiative, 2020d). At the preclinical phase, it proved highly active against many *Leishmania* species known to produce both visceral (Van den Kerkhof et al., 2018) and cutaneous leishmaniasis (Van Bocxlaer et al., 2019). In 2018, a phase I, blinded, randomized, single-dose trial was started to study the pharmacokinetics and tolerability of a single oral dose of DNDI-6148 in healthy male subjects (ISRCTN registry, 2020). The publication of the study results are expected in 2020.

The association between the University of Dundee, GlaxoSmithKline (GSK) and DNDi led to identification of GSK3186899/DDD853651 and GSK3494245/DDD1305143 as potential candidates to treat visceral leishmaniasis (Drugs for Neglected Diseases initiative, 2020f). Both compounds show a favorable pharmacokinetic profile and similar activity to the frontline drug miltefosine in animal models of visceral leishmaniasis (Wyllie et al., 2018, 2019; Thomas et al., 2019). *In vitro* studies indicate that GSK3186899/DDD853651 main mechanism can be attributed to the inhibition of the cdc-2-related kinase 12 (CRK12) of the parasite (Wyllie et al., 2018). Further studies confirmed that GSK3494245 inhibit the chymotrypsin-like activity in the *Leishmania donovani* proteasome (Wyllie et al., 2019). In 2019, GSK3186899 entered a double-blind study started to assess its safety, tolerability, and pharmacokinetic profile in healthy humans (ClinicalTrial.gov NCT03874234, 2020), but the study was recently suspended by GSK. By the end of this year, a phase I trial will be developed to assess the efficacy and safety of GSK3494245 (ClinicalTrials.gov NCT04504435, 2020).

Novartis and the University of Dundee have reported a selective proteasome inhibitor with efficacy in murine models of visceral and cutaneous leishmaniasis, LXE408 (Nagle et al., 2020), which is currently undergoing phase I human clinical trials (Drugs for Neglected Diseases initiative, 2020b).

Table 3 summarizes the current therapeutic options for leishmaniasis, as well drugs that have or are undergoing clinical trials.

**Table 3:** The therapeutic scenario for leishmaniasis, including WHO-recommended drugs, and current clinical trials.

**Drugs**	**Stage of the disease**	**Administration via**	**Adverse reactions**	**References**
**Drugs used to treat cutaneous and visceral leishmaniasis recommended by WHO**				
Amphotericin B, AmBD and liposomal amphotericin B, amphotericin B lipid complex, and amphotericin B colloidal dispersion 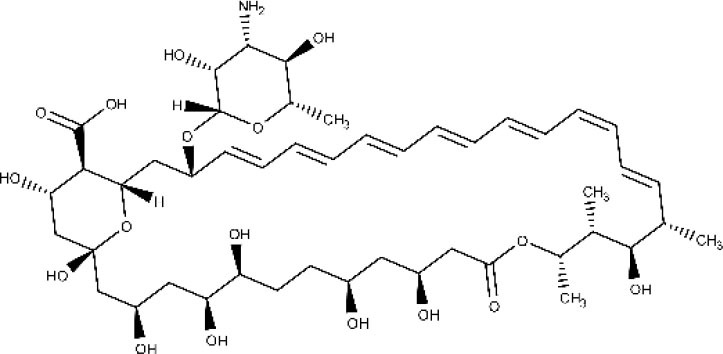	Visceral leishmaniasis	IV	Nephrotoxicity, also myocarditis and death. The liposomal, lipid and colloidal formulations show fewer side effects (rigors and chills)	(Freitas-Junior et al., 2012; ClinicalTrials.gov NCT0265679, 2020; World Health Organization, 2020d)
Pentavalent antimonials meglumine antimoniate and sodium stibogluconate 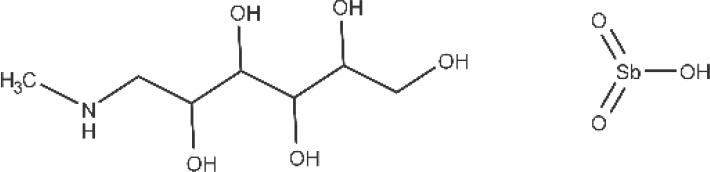	Visceral leishmaniasis and cutaneous leishmaniasis	Intravenous Intramuscular	Common side effects are anorexia, vomiting, nausea, abdominal pain, malaise, myalgia, arthralgia, headache, metallic taste and lethargy. High cardiotoxicity, pancreatitis, nephrotoxicity, hepatotoxicity; high treatment failure (up to 65% in major epidemic areas)	(Sundar et al., 2000; World Health Organization, 2020d)
*meglumine antimoniate* 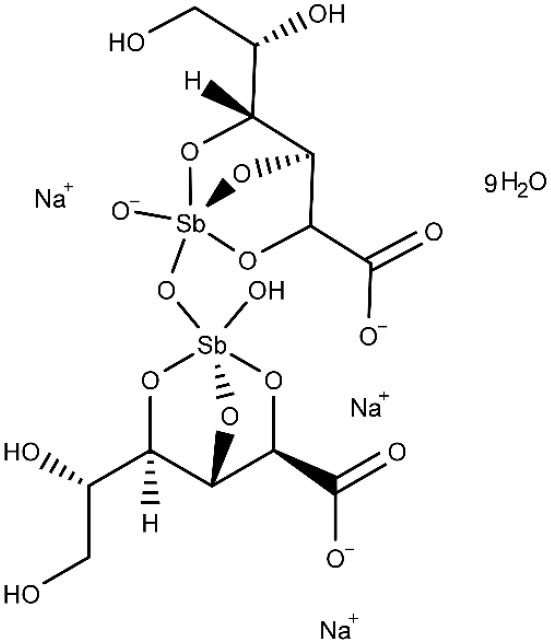				
*sodium stibogluconate*				
Paromomycin sulfate 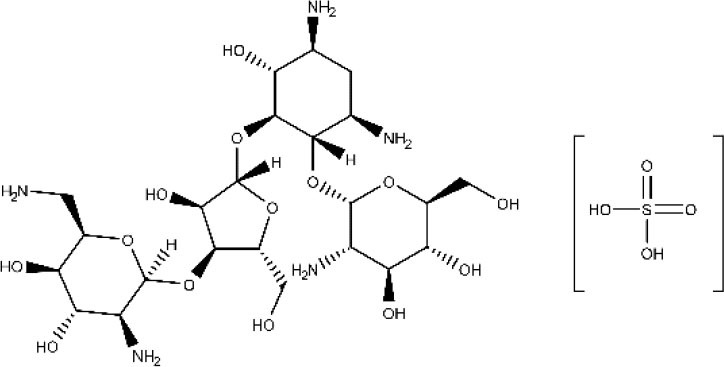	Visceral and cutaneous leishmaniasis	Intravenous Intramuscular Topical	Gastrointestinal symptoms including nausea, vomiting, diarrhea, and abdominal discomfort. Nephrotoxicity and ototoxicity are rarely produced	(Ben Salah et al., 2013; ClinicalTrials.gov NCT01140191, 2020; World Health Organization, 2020d)
Miltefosine 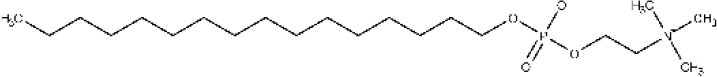	Visceral and cutaneous leishmaniasis	Oral	Gastrointestinal symptoms, nephrotoxicity, hepatotoxicity, teratogenicity	(Dorlo et al., 2012; ClinicalTrials.gov NCT03129646, 2020; World Health Organization, 2020d)
Pentamidine isethionate 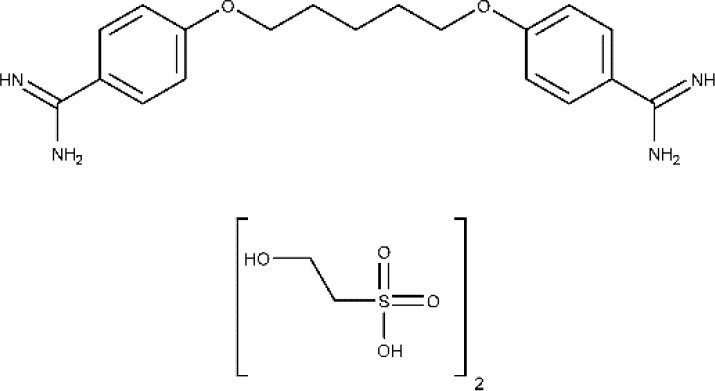	Visceral and cutaneous leishmaniasis	Intravenous Intramuscular	Diabetes mellitus, severe hypoglycemia, shock, myocarditis, and renal toxicity; limit its use	(ClinicalTrials.gov NCT02919605, 2020; World Health Organization, 2020d)
**Undergoing clinical trials**				
Miltefosine + liposomal amphotericin B + paromomycin	Post Kala Azar Dermal Leishmanioid	Oral (miltefosine) Intravenous (liposomal amphotericin) Intramuscular (paromomycin)	Not reported yet	(ClinicalTrials.gov NCT03399955, 2020; Drugs for Neglected Diseases initiative, 2020g)
Miltefosine + paromomycin	Visceral leishmaniasis	Oral	Not reported yet	(ClinicalTrials.gov NCT03129646, 2020)
Miltefosine + paromomycin	Cutaneous leishmaniasis	Oral (miltefosine) Topical (paromomycin)		(ClinicalTrials.gov NCT03829917, 2020)
DNDI-0690 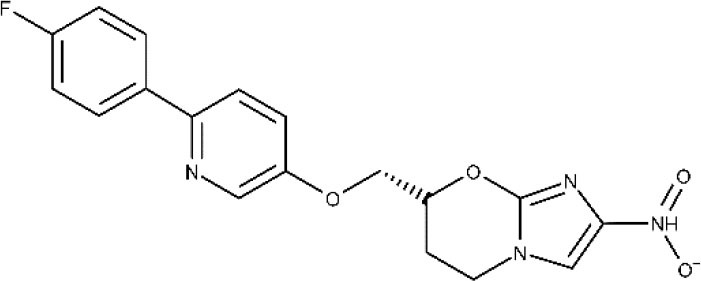	Visceral and cutaneous leishmaniasis	Oral	Not reported yet	(ClinicalTrials.gov NCT03929016, 2020)
DNDI-6148 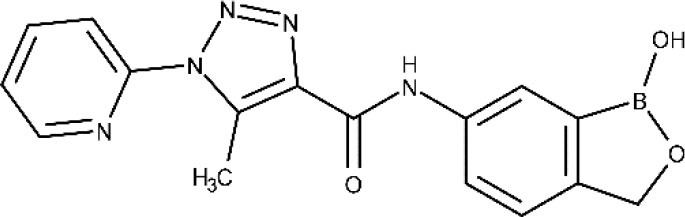	Visceral and cutaneous leishmaniasis	Oral	Not reported yet	(ISRCTN registry, 2020)
GSK3186899/DDD853651 (suspended) 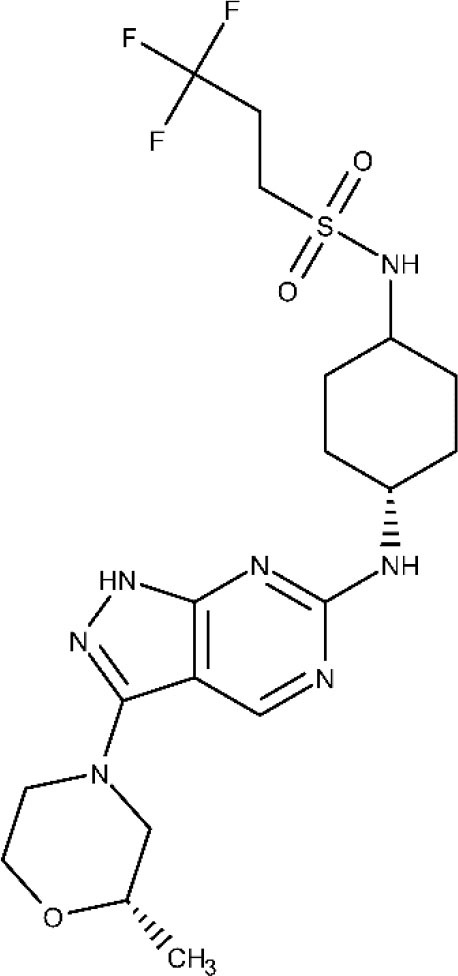	Visceral leishmaniasis	Oral	Not reported yet	(ClinicalTrials.gov NCT03874234, 2020)
GSK3494245/DDD1305143 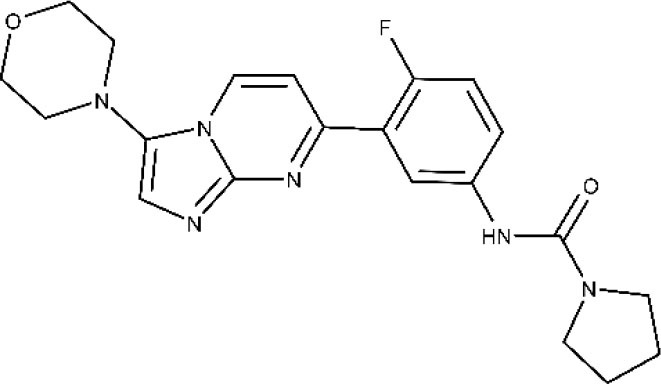	Visceral leishmaniasis	Oral	Not reported yet	ClinicalTrials.gov Identifier: NCT04504435
	Visceral and cutaneous leishmaniasis	Oral	Not reported	https://dndi.org/wp-content/uploads/2020/06/DNDi-June2020-RDPortfolio.pdf

### Lipid Nanosystems Encapsulating Approved Drugs

Leishmaniasis is so far the trypanosomatid-caused condition that has received more attention in the field of pharmaceutical lipid nanocarriers, probably due to the facts that one of the approved drugs against leishmaniasis (amphotericin B) is already available in liposomal formulation, and what is more, cutaneous leishmaniasis may at least be partially treated with topical medications. On the other hand, the predominant role of macrophages in the sequestration of circulating nanocarriers (Baboci et al., 2020) coupled with their importance on *Leishmania* infection establishment and the persistence of the parasite inside the host (De Menezes et al., 2017; Soulat and Bogdan, 2017; Holzmuller et al., 2018) may have contributed to the development of drug nanodelivery systems for this disease. Predictably, then, a substantial fraction of the reported nanosystems correspond to amphotericin B drug delivery systems. Noteworthy, leishmaniasis is also the trypanosomatid-caused infection for which the widest diversity of lipid nanocarriers has been investigated.

Because macrophages play a crucial role in leishmaniasis (they take part of the immune response against the parasite, but they also constitute the final host cells for its proliferation) (Tomiotto-Pellissier et al., 2018), many researchers have designed macrophage-directed antileishmanial drug delivery systems. Gupta et al. (2007) formulated amphotericin B in trilaurin emulsomes stabilized by soy phosphatidylcholine and targeted with O-palmitoyl mannan, a macrophage-specific ligand. The antileishmanial performance was tested. The antileishmanial activity was tested both *in vitro* and *in vivo*, demonstrating that the decorated emulsomes outperformed both non-decorated drug-loaded emulsomes and the free drug. Similar results were later observed in mice by Veerareddy et al. (2009) using mannose-decorated lipid nanospheres, comparing their efficacy with Fungizone. The decorated nanosystem achieved better performance than Fungizone or the non-decorated formulation. Additionally, mice treated with the encapsulated drug show no elevation in serum glutamate pyruvate transaminase, alkaline phosphatase, urea, and creatinine levels and increased bioavailability in comparison with Fungizone. Remarkably, the targeted system was found to distribute more rapidly to the liver and the spleen. Rathore et al. (2011) proposed a formulation of amphotericin B mannosylated liposomes for the active targeting to the reticular endothelial system. They compared the drug release and biodistribution (in a hamster model of infection) of the free drug, undecorated liposomes, and mannosylated liposomes. At 24 h, comparable but slightly lower percentage of drug release were observed for mannose-coupled liposomes, an expected behavior having in mind the additional diffusional layer present in the decorated carriers. The mannosylated formulation displayed the highest reduction in parasite load, and it was confirmed that the active targeting strategy led to a comparative reduction of circulating liposomes with the concomitant rise in drug increase in liver and spleen. Mannosylated nanomicelles have also been proposed to deliver combination therapy of amphotericin B and doxorubicin for visceral leishmaniasis (Wei et al., 2020).

Using nanoprecipitation followed by sonication, Patel and Patravale (2011) obtained amphotericin B SLN conceived for oral administration (AmbiOnp). The effects of several processes (probe sonication, dialysis cycles, freeze drying, and reconstitution) on the physicochemical features of the nanoparticles (such as particle size, dispersion and entrapment efficiency) were examined. Untreated SLN displayed a relatively high mean diameter (about 350 nm) and considerable dispersion; both were substantially reduced by sonication and dialysis. Drug loading significantly increased the mean diameter of the SLN. A single-dose acute toxicity study suggested the (acute) safety of AmbiOnp orally. Pharmacokinetic studies in rat using a non-compartmental approach suggested and enhanced relative bioavailability of AmbiOnp in comparison to a free drug solution (including a substantial increment in the elimination half-life, the time to reach the concentration peak in plasma, and the total area under the plasma concentration–time curve), although additional time points in the terminal phase of the concentration–time curve should have been obtained for a more accurate estimation of most of the estimated pharmacokinetic parameters. A subsequent study with the same formulation and an improved sampling schedule confirmed, however, that oral AmbiOnp presented increased half-life and total area under the concentration–time curve in comparison to intravenous Fungizone (Chaudhari et al., 2016).

Singodia et al. (2011) reported the obtention of alginate-coated amphotericin B–loaded lipid nanoparticles, hypothesizing that activation of macrophages by alginate would synergize the antileishmanial effects of the drug. The lipid nanoparticles without coating presented a mean particle size of 108 nm, whereas alginate coating increased the mean size to 134 nm and reversed the zeta potential from +28.4 to −19.8 mV. Moderate entrapment efficiency was observed. Inhibition of intramacrophage amastigotes was significantly higher compared to non-coated drug-loaded lipid nanoparticles. Very similar results were documented by Jain et al. (2014) who prepared chitosan-coated SLN loaded with amphotericin B for immunoadjuvant chemotherapy of leishmaniasis. Besides enhanced efficacy in comparison to Fungizone and AmBisome, the authors demonstrated the improved safety profile of the developed formulation in acute toxicity studies.

Lipid–polymer antileishmanial hybrid nanoparticles were designed by Asthana et al. (2015) composed of a poly(d,l-lactide-co-glycolide) core and a stearylamine shell and loaded with amphotericin B. Stearylamine was selected as the lipid component because of its immunostimulant activity and because it acts as a ligand for stearylamine pattern recognition receptors present on the macrophage surface. The stearylamine shell increased the size of the nanoparticles and reverted their charge from negative to positive. The particles used for subsequent studies were about 175 nm. The reported system displayed a very attractive profile, including sustained drug release, reduced erythrocyte and macrophage toxicity, enhanced macrophage uptake, higher accumulation in the spleen and the liver, minima distribution in kidneys, considerable antileishmanial efficacy *in vitro* and *in vivo* against visceral leishmaniasis, and low levels of nephrotoxicity markers. The authors indicated that macrophage pattern recognition receptors were involved in the uptake of the nanoparticles and that the positive charge of the lipid shell allows them to bind to the negatively charged macrophage surface, favoring adsorption mediated endocytosis. Another recently reported hybrid system loaded with both amphotericin B and paromomycin consisted of SLN modified with 2-hydroxypropyl-β-cyclodextrin as an oral matrix system against visceral leishmaniasis (Parvez et al., 2020). The reported formulation possesses a sustained drug release profile. *In vitro* studies verified the total cellular internalization of the modified SLNs with low cytotoxicity in macrophage cells within 24 h of incubation. Moreover, the nanosystem did not elicit hepatic or renal toxicity in mice. *In vitro* and *in vivo* studies showed improved efficacy on *L. donovani* intracellular amastigotes and significantly reduced the liver parasite burden in comparison to miltefosine.

A very interesting, novel approach for the targeted delivery of amphotericin B was devised by Kumar and Bose (2019), who implemented a “ghost cell” (nanoghost) strategy utilizing macrophage membrane-derived nanovesicles as a specific carrier for the drug. The nanoghost delivered the drug specifically to infected macrophages driven by antigenic identification of infected tissues associated to low toxicity toward healthy cells.

Less frequently, other approved drugs against leishmaniasis, besides amphotericin B, have been formulated in colloidal lipid systems. For instance, paromomycin SLN with good entrapment efficiency was reported by Ghadiri et al. (2011). Sometime later, Gaspar et al. (2015) reported the development of six paromomycin liposomal formulations whose biodistribution profiles revealed preferential targeting of the antibiotic to the spleen, liver, and lungs, relative to the free drug. Such observation translated into an augmented therapeutic effect in murine models of infection with *L. infantum* and improved safety profile. Positive results were also observed by Heidari-Kharaji et al. (2016), who prepared paromomycin-loaded SLN and tested their efficacy *in vivo* against *L. major*–infected mice. The parasite load in the footpad swelling was analyzed by real-time PCR, and the level of cytokines was also assessed. The study showed that the developed formulation was efficacious in killing the parasite and switching toward T_H_1 response.

da Gama Bitencourt reported the obtention of miltefosine-loaded lipid nanoparticles that enhanced the drug stability with reduced cytotoxicity in macrophages and diminished hemolytic potential, but also retaining the antiparasitic activity (da Gama Bitencourt et al., 2016). Interestingly, because of the amphiphilic nature of the drug, it acted as a powerful surfactant, and addition of increasing amounts of miltefosine reduced the mean particle size, from 144 nm (unloaded nanoparticles) to 40 to 65 nm.

Moosavian et al. (2019) obtained meglumine antimoniate-loaded liposomes containing stearylamine. Liposomal formulations enhanced the drug permeation compared with meglumine antimoniate cream. The liposomal formulation containing stearylamine proved more efficacious than drug-loaded liposomes without stearylamine. In a mice model of cutaneous leishmaniasis, liposomal groups presented smaller lesions compared to control.

### Lipid Nanosystems Encapsulating Non-approved Drugs

Lopes et al. (2012) obtained tripalmitin SLN encapsulating the potential antileishmanial dinitroaniline oryzalin. The nanoparticles were stabilized by mixed emulsifier molecules such as soy lecithin, sodium deoxycholate, and Tween 20. The cell viability experiments proved that the nanoencapsulation of the drug diminished its cytotoxicity. A subsequent study by the same group compared the safety and efficacy of oryzalin-loaded SLN and liposomes with those of the free drug (Lopes et al., 2014). As observed in other reviewed articles of the current section, the nanoformulations revealed diminished cytotoxicity and hemolytic activity in comparison with the free drug and without losing *in vitro* efficacy. Superiority of both nanocarriers on the reduction of parasitic burden in spleen and liver (compared with free oryzalin) was demonstrated in a mice model of visceral leishmaniasis. Kupetz et al. (2013) screened a number of colloidal systems to develop parental formulations of the poorly soluble paullon chalcone derivative KuRei300, including micelles stabilized with lecithin/bile salts, liposomes, supercooled smectic liquid crystal of cholesterol myristate nanoparticles, a triglyceride emulsion, and cubic phase nanoparticles.

Other lipid nanosystems have been proposed to exploit the antileishmanial activity of lipophilic compounds and mixtures of natural origin. Marquele-Oliveira et al. (2016) reported a nanodelivery system based on stearic acid SLN encapsulating the liposoluble lignan fraction of the South American plant *Ocotea duckei* Vattimo, which targets the *Leishmania* lysosome of macrophages. Physicochemical analysis revealed that the delivery system presented a core-shell architecture, and the correspondent dissolution studies revealed that the active components are released by a matrix diffusion-based kinetic mechanism. The loaded SLN displayed no toxicity to murine macrophages with an *in vitro* antileishmanial effect. Comparable results were reported by Want et al. (2017), who developed a liposomal artemisinin formulation. Nanoliposomal artemisinin proved superior performance compared to free artemisinin in a mice model of visceral leishmaniasis, with modulation of cell-mediated immunity toward protective T_H_1 type. Similarly, Kar et al. (2017) prepared NLC loaded with cedrol, one of the major sesquiterpenes obtained from the genus Cupressus). *In vivo* studies revealed that the antileishmanial effects of the orally administered nanoformulation were increased (in comparison with free cedrol and miltefosine) against wild type but also to drug-resistant strains of *L. donovani*. Sousa-Batista et al. (2017) reported the obtention of lipid-core nanoparticles made of capric/caprylic triglyceride, sorbitan monostearate (i.e., Span 60™), and poly(ε-caprolactone), for the oral delivery of quercetin and quercetin penta-acetate; the quercetin-loaded nanoparticles enhanced the oral efficacy of the drug in a model of cutaneous leishmaniasis (mice). Noteworthy, aspartate aminotransferase, alanine aminotransferase, or creatinine serum levels were not modified by the treatments, suggesting they had no liver or renal toxicity. Das et al. (2017) prepared ursolic acid–loaded NLC coated with chitosan oligosaccharides for the visceral leishmaniasis therapy, intended to deliver such active ingredient to macrophages following oral administration. The formulated NLC had nano sizes ranging from 104 to 143 nm, with high drug loading capacity and relatively good entrapment efficiency. The nanoformulation was highly efficient than the free drug against cellular amastigotes from a diversity of strains. It could also substantially suppress the parasite burden *in vivo*. Very recently, NLC containing the monoterpene carvacrol (which, despite promising antileishmanial activity, displays low water solubility, high volatility, and stability issues) has been described (Galvão et al., 2020). The highest encapsulation efficiency was achieved by using beeswax as solid lipid. The drug release from the NLC fitted to the Korsmeyer and Peppas, and Weibull models, suggesting a Fickian release mechanism. Carvacrol incorporation to the NLC resulted in diminished cytotoxicity in comparison to the free drug, also increasing its *in vitro* antileishmanial efficacy (amastigotes). The encapsulation also led to increased elimination half-life in rats.

Recently, Smith et al. (2018) described high-loading self-nanoemulsifying systems for the oral delivery of the poorly soluble antiprotozoal hydroxynaphthoquinone buparvaquone. This self-emulsifying system showed an improved oral bioavailability compared to aqueous dispersions, which translated into an increase area under the plasma concentration–time curve. It demonstrated potent *in vitro* efficacy, and it almost completely suppressed parasite replication in the spleen, whereas it also inhibited the parasite replication in the liver. Mazur et al. (2019) devised beeswax nanoparticles containing copaiba oil to encapsulate diethyldithiocarbamate, which has previously shown excellent leishmanicidal effect. The nanoformulation decreased the cytotoxic effects of the drug against macrophages, which led to an almost 2-fold increase in the selectivity index.

## Conclusions

The reviewed literature shows that the state of the art of lipid nanodelivery systems in the field of human trypanosomatid-caused diseases greatly varies, depending on the disease. Limited efforts have still been made in relation to drug nanocarriers for HAT or Chagas. In the case of HAT, the limited interest in pharmaceutical nanocarriers possibly responds to the favorable evolution of the epidemiological data in the last decade and the incorporation of novel, efficacious, convenient, and bioavailable options to the therapeutic arsenal. Regarding Chagas disease, there are still not enough or convincing data that suggest that the use of nanodevices could help overcoming the limitations of the currently (and extremely limited) available medications. In any case, the reviewed articles show that most of the reported lipid nanosystems for HAT or Chagas correspond to delivery systems from previous generations (prominently, non-targeted liposomes and SLN). In other words, the potential contribution of state-of-the-art lipid nanocarriers, including functionalized and hybrid systems, has still to be explored. The most relevant challenges here might be the delivery of effective levels of the drugs to poorly irrigated/accessible tissues, targeted delivery to the most affected organs (which could contribute to improved safety and tolerability), and the enhanced effect on dormant parasites.

The scenario is substantially different in the case of leishmaniasis, where a large variety of lipid systems with distinctive architectures and functionalities have been successfully tested at the preclinical level, including macrophage-targeted systems with enhanced parasiticidal effects in affected organs such as liver or spleen and immunostimulant hybrid systems. Either nanoencapsulating already approved or experimental drugs, the outcome of those studies with a focus on lipid nanosystems consistently includes sustained drug release, reduced cytotoxicity and liver and renal toxicity, increased safety and efficacy, and improved bioavailability.

Multiple factors can possibly explain the different scenarios described across diseases, including historical and pathophysiological aspects. Colloidal drug delivery systems (liposomal amphotericin B) are already available to treat leishmaniasis. The disease includes cutaneous presentations that can be treated systemically, but also in combination with topical formulations. Particularly, the macrophages have a relevant role in the establishment and evolution of the infection, and as we know, they are likely to be targeted by pharmaceutical nanocarriers. A considerable proportion of the studies linked to lipid systems in leishmaniasis focus on the oral delivery of poorly soluble/poorly bioavailable drugs, which includes a diversity of lipophilic natural products. Lipid nanocarriers thus constitute a valid alternative to exploit the rich chemical biodiversity and expand the therapeutic options for neglected conditions.

Besides their already discussed advantages (e.g., biocompatibility and biodegradability), other possible reasons might explain the relative abundance of studies linked to the development of lipid nanocarriers for the treatment of trypanosomatid-caused conditions. Among them is the suitability of these delivery systems to load comparatively high amounts of lipophilic agents. It should be considered that, in the case of parasitic diseases, the therapeutic agents must often access to the parasite intracellular space. Furthermore, some parasite reservoirs might be found in deep tissues (poorly irrigated tissues such as adipose tissue or tissues separated from circulation by specialized barriers, e.g., the brain). In any case, lipophilic chemotherapies would be often required to provide an extensive drug distribution and circumvent the correspondent biological barriers. These facts explain why many therapeutic agents for parasitic diseases do display a significant lipophilicity (in fact, they are one of the therapeutic categories whose members often violate Lipinski rules).

The route of administration and the costs are almost key factors when formulating active ingredients, but that is especially true when dealing with therapeutics for neglected conditions, as the convenience of the dosing forms and the cost of the therapeutic intervention are particularly relevant. Enhancing the efficacy–safety balance of already known drugs by encapsulating them within state-of-the-art nanovehicles could provide affordable solutions for the treatment of neglected conditions. Furthermore, the recent reports on quiescent or dormant stages of the parasite that cause the (today) most challenging human trypanosomatid-caused disorders (leishmaniasis and Chagas disease) may at least partially explain drug failure. It has been suggested that therapeutic benefits might thus be achieved with extended treatments; if so, pharmaceutical carriers enhancing tolerability could be more advantageous than ever. All things considered, improved safety could contribute to treatment adherence (a fundamental aspect in the field of infectious diseases, both from individual and public health perspective) and to the design of well-tolerated extensive dosing plans.

Despite its continuous and vertiginous progress, nanobiotechnology is still an emerging field, and many technological and regulatory challenges are to be faced before massive adoption within the pharmaceutical industry. Individual and environmental toxicological aspects and accurate and standardized evaluation of their pharmacokinetic profile are also to be solved. Lipid-based systems, because of their biocompatible nature, appear as a reasonable option to address some of these issues, whereas stability and scaling-up cost are possibly among their major disadvantages.

Finally, we would like to underline the need to explore last-generation pharmaceutical nanocarriers as vehicles for the treatment of trypanosomatid-caused diseases, disorders that in most cases have been only addressed preclinically using early generations of lipid-based systems. Unfortunately, for the time being, the development of therapeutic options involving last-generation technologies possibly collides with the necessity to achieve affordable solutions for neglected conditions.

## Author Contributions

All authors listed have made a substantial, direct and intellectual contribution to the work, and approved it for publication.

## Conflict of Interest

The authors declare that the research was conducted in the absence of any commercial or financial relationships that could be construed as a potential conflict of interest.
